# The association between ambient temperature and mortality of the coronavirus disease 2019 (COVID-19) in Wuhan, China: a time-series analysis

**DOI:** 10.1186/s12889-020-10131-7

**Published:** 2021-01-11

**Authors:** Gaopei Zhu, Yuhang Zhu, Zhongli Wang, Weijing Meng, Xiaoxuan Wang, Jianing Feng, Juan Li, Yufei Xiao, Fuyan Shi, Suzhen Wang

**Affiliations:** 1 Department of Health Statistics, School of Public Health, Weifang Medical University, No. 7166 Baotong West Street, Weifang, 261053 People’s Republic of China; 2 Department of Child and Adolescent Psychiatry, Psychotherapy, and Psychosomatics, Center for Psychosocial Medicine, University Medical Center Hamburg-Eppendorf, Martinistraße 52, W 29, 20246 Hamburg, Germany; 3 School of Public Health, Cheeloo College of Medicine, Shandong University, Jinan, 250012 People’s Republic of China; 4 School of Life Sciences and Technology, Weifang Medical University, No. 7166 Baotong West Street, Weifang, 261053 People’s Republic of China

**Keywords:** COVID-19, Ambient temperature, Mortality, Distributed lag non-linear model, Negative correlation

## Abstract

**Background:**

The COVID-19 has caused a sizeable global outbreak and has been declared as a public health emergency of international concern. Sufficient evidence shows that temperature has an essential link with respiratory infectious diseases. The objectives of this study were to describe the exposure-response relationship between ambient temperature, including extreme temperatures, and mortality of COVID-19.

**Methods:**

The Poisson distributed lag non-linear model (DLNM) was constructed to evaluate the non-linear delayed effects of ambient temperature on death, by using the daily new death of COVID-19 and ambient temperature data from January 10 to March 31, 2020, in Wuhan, China.

**Results:**

During the period mentioned above, the average daily number of COVID-19 deaths was approximately 45.2. Poisson distributed lag non-linear model showed that there was a non-linear relationship (U-shape) between the effect of ambient temperature and mortality. With confounding factors controlled, the daily cumulative relative death risk decreased by 12.3% (95% CI [3.4, 20.4%]) for every 1.0 °C increase in temperature. Moreover, the delayed effects of the low temperature are acute and short-term, with the most considerable risk occurring in 5–7 days of exposure. The delayed effects of the high temperature appeared quickly, then decrease rapidly, and increased sharply 15 days of exposure, mainly manifested as acute and long-term effects. Sensitivity analysis results demonstrated that the results were robust.

**Conclusions:**

The relationship between ambient temperature and COVID-19 mortality was non-linear. There was a negative correlation between the cumulative relative risk of death and temperature. Additionally, exposure to high and low temperatures had divergent impacts on mortality.

## Background

Infectious disease is an old and heavy term. From smallpox, plague, cholera, malaria, etc. in the early days of human civilization, to the Ebola hemorrhagic fever (EHF) and the Acquired Immune Deficiency Syndrome (AIDS) in the 1970s and 1980s, all of them have caused a large number of deaths, disabilities and economic losses [1, 2]. It can be said that the history of human development is a history of humans fighting infectious diseases [3]. Since the twenty-first century, viral respiratory infections, especially that coronavirus-associated pneumonia, have become a severe public health crisis [4]. The Severe Acute Respiratory Syndrome in 2002 [5, 6] and Middle East Respiratory Syndrome in 2012 are typical of these diseases [7, 8]. More recently, the COVID-19 proved that the occurrence of a new and dangerous infectious disease could monopolize governmental activities, cause fear and hysteria, and get a significant impact on the free life of people throughout the world [9].

COVID-19 has attracted attention due to the report of unexplained pneumonia in Wuhan, China [10, 11]. It was caused by SRAS-COV-2 infection [12], and subsequently spread to many other parts of the world through global travel. At present, COVID-19 outbreaks have occurred in South Korea, Italy, the United States of America, and other countries, and has been defined as a global pandemic [13]. According to incomplete statistics, as of April 30, approximately 3.2 million cases have been confirmed worldwide, with approximately 224,000 deaths. The number of global confirmed cases and deaths continued to increase. The intermittent emergence and outbreaks of coronaviruses remind us that they pose a severe threat to global health [14].

This epidemic reminds us of the public health crisis that was also caused by coronavirus seventeen years ago. At present, there was clear evidence that the characteristics of this outbreak are similar to those of the 2002 SRAS epidemic [15]. According to the previous research reports, the age, underlying health conditions, and environment were the significant factors determining the spread speed and fatality rate of SARS [5, 16]. Therefore, we can guess that the above factors may be closely related to COVID-19. It is gratifying that recently some prospective studies [17–19] provide an association between factors (age, basic health situation, and virus transmission speed) and mortality of COVID-19. However, to our best knowledge, some relationship between environmental factors, including meteorological factors and the death risk of COVID-19 patients remain unknown, might need further investigation. Recently, a new study described the relationship between meteorological factors and the death toll of COVID-19. Still, the study hypothesized that there was a non-linear relationship between ambient temperature and the death toll of COVID-19, and analyzed the relationship between linear lag of temperature and the death toll of COVID-19 [20]. However, this linear delayed effect hypothesis seems to contradict some of the previous general research results. Because a lot of previous studies have confirmed that there was a non-linear delayed effect between temperature and death [21–26]. Furthermore, there was also methodological evidence that it is dangerous and unwise to use the generalized additive model (GAM), with the delayed structure of linear effects, to analyze the relationship while ignoring the non-linear delayed effects. Because this method ignores the non-linear delay effect, thus concealing the real relationship between environmental factors and death [27]. In other words, if the linear correlation assumption is not met, the linear model may be not reliable to estimate the genuine relationship between temperature and death.

In fact, relevant studies have adopted a more appropriate model — distributed lag non-linear model (DLNM) to deal with this situation [28]. Therefore, this model was worth recommended to analyze the non-linear delayed effects between COVID-19 mortality and temperature. Besides, there has been robust evidence that the impact of extreme temperatures needs to be taken into account when focusing on the relationship between average temperature and death, as they may cause unexpected influence on death [29, 30]. Therefore, analyzing the temperature specific effects between extreme temperatures and COVID-19 mortality was undoubtedly a reasonable choice.

Therefore, a time-series study based on the distributed lag non-linear model was conducted to examine the influence of ambient temperature on mortality outcomes in COVID-19 patients, which can capture the delayed effects of temperature and identify extremely temperature-mortality risks. Additionally, the overall cumulative exposure-response between ambient temperature and COVID-19 death with delayed effects were also analyzed.

## Methods

### Study area

**Fig. 1:**
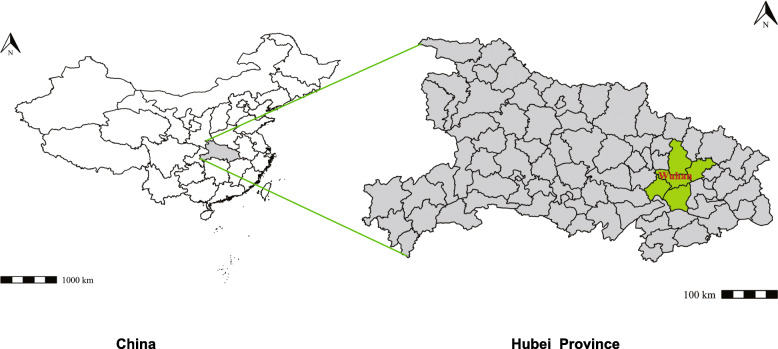
Location of Wuhan in Hubei Province, China. The green area indicates the location of Wuhan City, which situated in the east of Hubei Province, People’s Republic of China. The map depicted in Fig. 1 was built in the map software packages in R 3.5.3, which was open access. Additionally, maptools, and mapproj software packages in R 3.5.3 were also used to draw Fig. 1

Figure 1 shows the geographic position of Wuhan city in the east of Hubei Province, which is located where the Yangtze River joins its largest tributary, the Han River. Wuhan covers an area of about 8569.15 km^2^, and the registered population was 11.212 million in 2019. Wuhan is located between latitude 29°58′–31°22′N and longitude 113°41′–115°05′E, which has a subtropical monsoon humid climate with an annual average temperature of 15.8–17.5 °C and the average yearly rainfall of 1150–1450 mm. The city has four distinct seasons, with cold, wet winter and hot, humid summer. Also, Wuhan is an important science and education base and transportation hub (http://www.wh.gov.cn/zjwh/).

### Data collection

We collected the data on the number of daily new COVID-19 deaths, ambient temperature, humidity, air quality index (AQI), migration scale index (MSI) and urban travel index (UTI) from January 10 to March 31, 2020, in Wuhan.

The urban MSI can show the status of population mobility and reflect the scale of the population migration from a city in a unit time [31]. UTI is an another travelling index, which can be used to measure the population density of inner-city travel. The data of MSI and UTI were obtained from the Baidu map migration platform in the people’s Republic of China (https://qianxi.baidu.com/).

The daily COVID-19 death toll was obtained from the websites of the National Health Commission of the People’s Republic of China. The daily average temperature and humidity data were obtained from the Meteorological science data sharing service of the People’s Republic of China (http://data.cma.cn/site/index.html) and AQI data from air quality monitoring analysis online platform of the People’s Republic of China (https://www.aqistudy.cn/historydata/).

### Statistical analysis

The semi-parametric generalized additive model was used to assess the relationship between environmental epidemiology exposure and death [32–34]. The influence of the latency period of COVID-19 and time of admission were also considered and put into the model. The average incubation period is 5.2 days (range: 2–7 days) [10, 35], and the median time of admission was about 10 days [36]. Since the coincidence of the delayed effect of latency period and time of admission in the relationship between temperature and death [37], the temperature delayed period of this study was set to 15 days.

Relative to the total population, daily COVID-19 deaths were defined as a small probability event, which follows the Poisson distribution [38]. The influence of air temperature on health usually has a delayed effect, and the relationship is not linear [26, 39, 40]. In this study, the Poisson function was used as the connection, and the generalized additive model (GAM) was used as the core model. The distributed lag non-linear model (DLNM) was used to analyze the time-series data to estimate the influence of temperature on the death of COVID-19 and the delayed effect. The temperature was included in the form of a cross-basis to estimate its impact on COVID-19 in both variable levels and time lag dimensions. Meanwhile, in order to balance the influence of other factors, relative humidity and AQI were incorporated into the model with the natural cubic spline function, and the model was finally established as follows:

$$ \mathrm{Log}\left[\mathrm{E}\left({\mathrm{y}}_{\mathrm{t}}\right)\right]=\upalpha +\upbeta {\mathrm{Temperature}}_{\mathrm{t},\mathrm{l}}+\mathrm{NS}\left({\mathrm{Humidity}}_{\mathrm{t}},\mathrm{df}\right)+\mathrm{NS}\left({\mathrm{AQI}}_{\mathrm{t}},\mathrm{df}\right)+\mathrm{NS}\left(\mathrm{time},\mathrm{df}\right)+\mathrm{NS}\left({\mathrm{MSI}}_{\mathrm{t}},\mathrm{df}\right)+\mathrm{NS}\left({\mathrm{UTI}}_{\mathrm{t}},\mathrm{df}\right) $$

*y*_*t*_ is the number of death cases of COVID-19 on day *t*, which follows the Poisson distribution of *E* (*y*_*t*_). α is the constant term of the model, *Temperature*_*t,l*_ is the cross basis of temperature and delay time, *β* is its coefficient. *NS* is the natural spline function. Adjust variables such as relative humidity and AQI. *Humility*_*t*_ is day *t* relative humidity. AQI_*t*_ is day *t* air quality index. MSI_*t*_ is day *t* migration scale index and UTI_*t*_ is day *t* urban travel index. *l* and *df* are the delay days and degrees of freedom, respectively, and *time* is the date of day *t*.

Sensitivity analyzes were performed to assess the robustness of the model. First, we assessed cumulative exposure using the mean temperatures of successive 0–14, 0–15, and 0–16 days, respectively. Secondly, we apply different degrees of freedom (6–8) to time to adjust the unmeasured time-varying confounding. Finally, the robustness of the results was evaluated by removing the daily average AQI or daily average relative humidity, respectively.

The tests were two-sided, and values of *p* < 0.05 were considered statistically significant. All statistical analysis and graphic plotting were conducted with the free software environment—R (version 3.5.3, R Development Core Team, March 2020). Specifically, we used the software packages ‘mgcv’ and ‘dlnm’ to examine the effects of non-linear delayed effects. Besides, the ‘dlnm’ package was also used to construct a cross-basic matrix for mortality and temperature. All the software packages used above are publicly available on the R Comprehensive Archive Network (CRAN) (https://cran.r-project.org/).

## Results

### Descriptive analysis

**Fig. 2:**
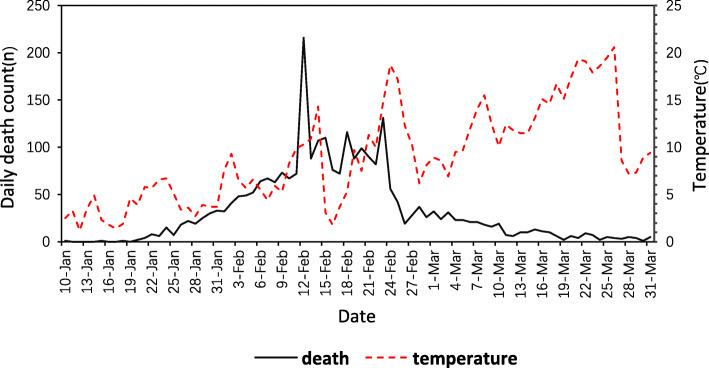
The daily distribution of daily death count and mean temperature in Wuhan from 1 January 2020 to 31 March 2020

By March 31, 2020, a total of 50,007 cases and 2553 deaths were reported in Wuhan, accounting for 73.75% of the cumulative COVID-19 deaths in China. The case fatality rate was 5.10%. Table 1 summarized the characteristics of the number of deaths, AQI, and relative humidity of COVID-19 in Wuhan from January 10 to March 31, 2020. The maximum number of deaths on COVID-19 was 216, and the minimum was 0. The daily average temperature was 9.0 °C, and the maximum temperature was 20.6 °C. Figure 2 shows the daily distribution of the number of deaths and the mean temperature on COVID-19. The results showed that the temperature was gradually increasing, and the death number of COVID-19 gradually increased and then decreased in Wuhan.

**Table 1:** Statistics of daily death cases and mean temperature in Wuhan

Variable	N_0_ (day)	Mean	SD	Min	P (25)	Median	P (75)	Max
COVID-19 Mortality Counts	82	32.4	38.8	0	5.0	19.0	49.7	216.0
Temperature (°C)	82	9.0	5.1	1.2	4.8	8.4	12.3	20.6
Relative humility (%)	82	81.2	7.9	59.0	76.7	82.0	87.0	97.0
AQI	82	63.6	26.9	20.0	41.5	60.0	77.2	142.0
MSI	82	1.1	1.4	0.3	0.3	0.4	1.25	4.8
UTI	82	1.4	1.4	0.6	5.2	0.7	0.6	1.3

Note: *N*_*0*_ the number of days, *SD* standard deviation, *Min* the minimum value, *Max* the maximum value, *AQI* air quality index, *MSI* migration scale index, *UTI* urban travel index, *P (25)* upper quartile, *P (75)* lower quartile

### Association of temperature lag and COVID-19 mortality

**Fig. 3:**
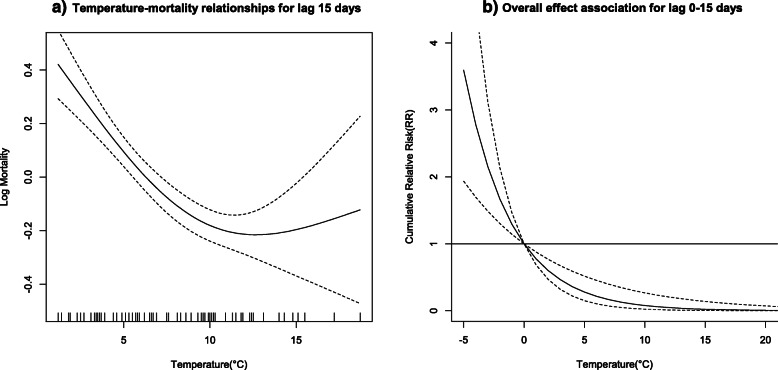
Temperature-mortality relationships (**a**) and death cumulative RR for daily mean temperature at lag0–15 days (**b**)

Using the Poisson generalized additive model for time series analysis, the correlation between the daily log of COVID-19 mortality and temperature and 15 lag days was visualized (Fig. 3a). From the figure, the correlation is U-shaped, and the delayed effect is non-linear. Besides, compared with the average temperature, when the temperature is lower, COVID-19 mortality is higher. As the ambient temperature increases, the Log (mortality) of COVID-19 patients due to temperature initially decreases rapidly and then slowly increases.

Figure 3b displays the overall correlation between cumulative relative risk of COVID-19 death and temperature, which is L-shaped. A significant negative association was shown between the temperature and the daily risk of COVID-19 death, in other words, a 1.0 °C increase in temperature was associated with a 12.3% (95% CI [3.4, 20.4%]) reduction in daily cumulative relative risk of COVID-19 death. When the temperature was lower than 20.0 °C, the relative risk of death is approaching 0 while it was close to 20.0 °C. Overall, the cumulative relative death risk of COVID-19 decreased with increasing temperature.

**Fig. 4:**
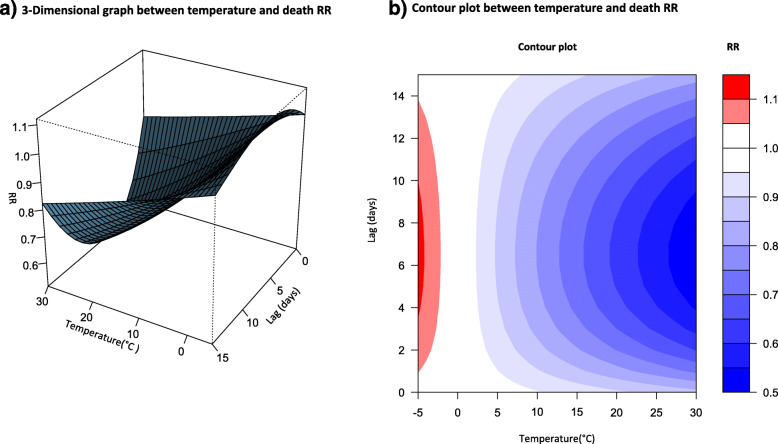
Relative risks of mortality by daily mean temperature along 15 lag days

**Fig. 5:**
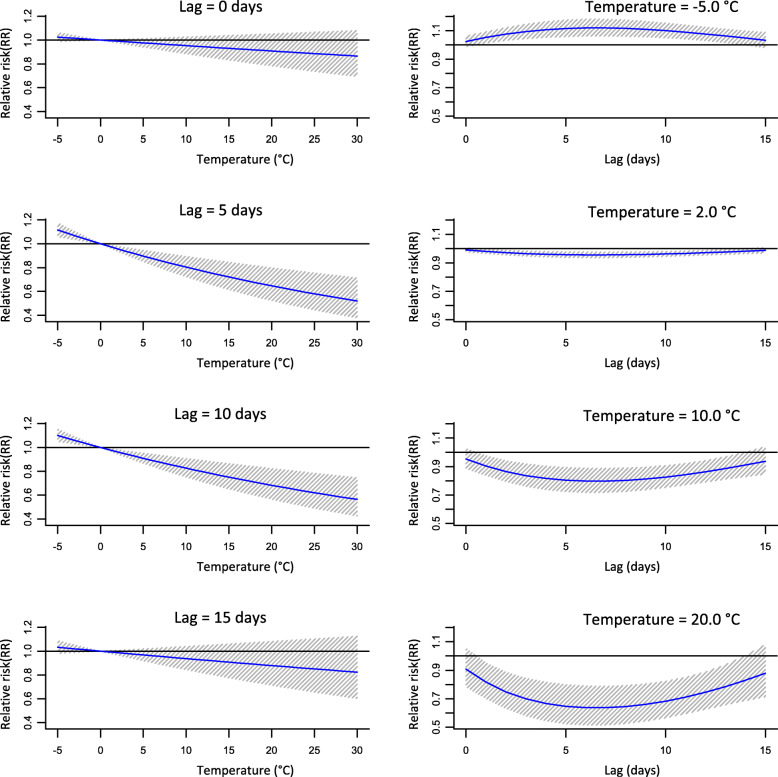
The relative risk of mortality by daily mean temperature at a specific lag day (0, 5, 10, 15 days) and temperatures (− 5.0, 2.0, 10.0, 25.0 °C)

Figure 4 shows the general pattern of the relative risk death as a function of temperature and lag, by showing a three-dimensional plots of relative death risk along with temperature and lag 15 days. Overall, the effect of temperature on the daily mortality risk of COVID-19 was non-linear, with higher temperatures leading to lower relative risk. Figure 5 shows the relative mortality for the lag-specific effects (0, 5, 10, 15 days) and temperatures-specific effects (− 5.0, 2.0, 10.0, 20.0 °C). The death risk of COVID-19 at low temperature presented acute and short-term effects, and it showed a trend of first strong and then weak, with the greatest risk occurring in 5–7 days of exposure. The delayed effects of the high temperature appeared quickly, then decrease rapidly, and increased sharply 15 days of exposure, the mortality risk of COVID-19 presented as acute and longer-term effects. Also, low temperatures had a shorter impact on the mortality risk of COVID-19 than high temperatures.

### Poisson generalized additive model

Temperature, humidity, AQI, MSI and UTI were incorporated into the final model. Especially for temperature, the distributed lag nonlinear structure was considered, the then the nonlinear lag period was set as 15 days, along with the degree of freedom of the long-term trend of time that was set as 7. After adjusting for humidity, AQI, MSI, and UTI, the relative risk of COVID-19 death decreased by 5.4% (95%CI [3.4, 6.9%]) for every 1 °C rise in average temperature (Table 2). Humidity, AQI, and MSI had no significant effect on COVID-19 deaths. In addition, for per 1-unit increase in UTI, the relative risk of COVID-19 death nearly doubled: 1.959 (95%CI [1.009, 3.804]).

**Table 2:** The effect of a one-unit increase in average temperature, relative humility, AQI, MSI and UTI on daily death cases of COVID-19

Factors	Relative Risk	95% CI	*p value*
Temperature (°C)	0.948	[0.931,0.966]	< 0.001
Relative humility (%)	0.994	[0.987,1.000]	0.054
AQI	0.999	[0.996,1.002]	0.534
MSI	0.642	[0.362,1.137]	0.120
UTI	1.959	[1.009,3.804]	0.042

Note: *AQI* air quality index, *MSI* migration scale index, *UTI* urban travel index, *CI* confidence interval

The temperature delayed period of the final model was set to 15 days

### Sensitivity analyses

Changing the time degree of freedom (6–8) could control long-term trends and seasonality. Some influencing factors were eliminated to obtain the adjustment model. In this study, relative humidity and AQI were eliminated, respectively. Table 3 shows that under the conditions of temperature lag0–14 days, lag0–15 days, and lag0–16 days, the average cumulative death effects (RR [95% CI]) of COVID-19 did not change significantly. Under the conditions of temperature lag14 days, lag15 days, and lag16 days, the COVID-19 death effects (RR [95% CI]) also did not change significantly. In conclusion, the model applied in this study were robust.

**Table 3:** Sensitivity analysis death RR [95% CI] of COVID-19 caused by temperature in Wuhan

Lag	Model 1	Model 2	Model 3	Model 4	Model 5
Lag14	0.978 [0.962,0.996]	0.974 [0.956,0.991]	0.972 [0.954,0.990]	0.973 [0.955,0.990]	0.969 [0.952,0.986]
Lag15	0.960 [0.943,0.977]	0.956 [0.936,0.972]	0.953 [0.934,0.971]	0.953 [0.935,0.971]	0.946 [0.929,0.964]
Lag16	0.977 [0.959,0.996]	0.975 [0.956,0.994]	0.975 [0.956,0.995]	0.973 [0.955,0.992]	0.962 [0.943,0.980]
Lag0–14	0.945 [0.863,1.034]	0.955 [0.870,1.049]	0.921 [0.832,1.021]	0.965 [0.888,1.064]	0.982 [0.907,1.063]
Lag0–15	0.878 [0.798,0.965]	0.877 [0.796,0.966]	0.848 [0.765,0.941]	0.891 [0.813,0.979]	0.907 [0.836,0.983]
Lag0–16	0.823 [0.742,0.913]	0.815 [0.733,0.906]	0.804 [0.723,0.895]	0.830 [0.750,0.920]	0.853 [0.780,0.932]

Note: *Model 1* long-term degree of freedom is 6, *Model 2* long-term degree of freedom is 7, *Model 3* long-term degree of freedom is 8, *Model 4* model excluding daily AQI, *Model 5* model excluding daily average relative humidity, *RR* relative risk, *CI* confidence interval, *Lag* the delayed effects situation of temperature, *Lag14* the COVID-19 death effects of the conditions of temperature lag 14 days, *Lag0–14* the COVID-19 average cumulative death effects of the conditions of temperature lag 14 days

## Discussion

There is no doubt that the outbreak of COVID-19 has caused enormous economic loss and health burden around the world. Under such circumstances, independent and robust scientific evidence will undoubtedly provide a powerful weapon to deal with this crisis. We believe that it is significant to clarify the relationship between ambient temperature and mortality of the COVID-19, not only in Wuhan but also in other epidemic areas in the world. In this study, we used a rigorous and scientific mathematical model to reveal the unique relationship between temperature and death caused by COVID-19, even if the association between death and temperature in non-communicable diseases has been established [40]. We hope that the research results can provide some methodological guidance for the response to this crisis.

DLNM model was verified to be a useful tool in this study to assess the non-linear relationship between ambient temperature and COVID-19 mortality on a daily basis, including properly evaluating the non-linear associations and cumulative death relative risks related to temperatures for lag days. The model figures out the non-linear and negative correlation between ambient temperature and COVID-19 mortality [26, 40, 41]. The increase in temperature could reduce the death risk of patients, and the relationship between temperature and death effect was U-shaped.

Our study found that the relationship between death risk of COVID-19 and low temperature was different from the high temperature. The low temperature effect on the death risk of COVID-19 is first enhanced and then weakened. With increasing of outdoor temperature, the death risk of COVID-19 is decreasing. The increase in temperature may reduce the lethal intensity of COVID-19, which is related to the increase of virus inactivation caused by high temperature [36, 42]. When the ambient temperature rose to around 10.0 °C and continued to rise, the temperature and the death risk of COVID-19 gradually decreased and then increased, which is consistent with the findings in non-communicable diseases [39]. When the temperature is getting higher and higher beyond the inflection, the death risk of combined diseases such as AIDS, diabetes and hypertension may also increase [23], which potentially increases the death risk of patients with COVID-19. Besides, the low temperature effects are acute and short-term [43], with the most considerable risk occurring in 5–7 days of exposure. High temperature mainly reflects the acute effect, and the maximum effect occurs on the day of temperature exposure, which is similar to some studies [44, 45].

The results of the study show that low temperature has a more significant impact on the death risk of COVID-19 than high temperature is consistent with a meta-analysis [46]. At low temperatures, deaths from respiratory illnesses are greatly affected. Exposure to low temperature in humans can cause cardiovascular stress, which is affected by factors such as peripheral blood vessel constriction, plasma cholesterol, plasma fibrinogen, red blood cell count, blood viscosity, and inflammatory response [47, 48]. These factors together lead to respiratory distress, thus contribute to the deterioration of COVID-19 patients. At high temperatures, the number of patients dying from chronic non-communicable diseases increased, which forms a potential competitive relationship, leading to a gentle change in the number of COVID-19 deaths directly attributable to temperature [40, 49].

Overall, the temperature was negatively correlated with the cumulative effect of COVID-19 deaths [24]. At low temperatures, the cumulative death risk of COVID-19 was higher. With the increase of daily average temperature, the delayed effects of temperature exposure in patients with COVID-19 decrease rapidly and show protective effects. This data indicates that the risk of death of COVID-19 patients gradually decreases due to the increase in ambient temperature. With the advent of summer, the COVID-19 patient population may benefit from the high temperature effect.

Sensitivity analysis showed that the results of this study were robust. Firstly, the distributed lag non-linear method can flexibly dig out the possible relationship between temperature changes and daily mortality and cumulative delayed effects. Although the model is involved with many parameters, our sensitivity analysis shows that the results are robust [49]. Secondly, during the analysis, we adjusted a group of potential confounding factors, including daily average temperature, relative humidity and AQI, and compared the model results after excluding relative humidity or AQI. Generally, our results were relatively robust.

Some limitations should be considered in interpreting our findings: Firstly, this is an ecologically designed study, and the use of environmental monitoring data may not accurately reflect actual personal exposure. Secondly, COVID-19 patients basically receive isolation treatment in the designated hospital, and the patients live in the closed space, so the relationship between the ambient temperature and death may be different from that of indoor temperature. Third, this study did not adjust the social and demographic factors such as age and economy, which may affect the population structure and mortality [9]. Fourth, individual basic disease information such as diabetes, hypertension and AIDS are not available on the websites of the National Health Commission of the People’s Republic of China, which will cause bias to our research results. Finally, in the process of treating and curing COVID-19 patients, clinical diagnosis and treatment guideline is continuously updated, and the impact from this inconstancy was not included in this study.

## Conclusion

Despite these limitations, this study found out the non-linear Negative correlation between ambient temperature and death in COVID-19 patients. Besides, it was made clear that low temperature can potentially increase the risk of death, while high temperature manifests reversely. However, high temperatures may increase the risk of death from other complications, which are worthy of further study. Altogether, this study may provide a beneficial reference for the setting of COVID-19 clinical isolation and treatment environment.

## Data Availability

The datasets analyzed in this study were publically available. The daily COVID-19 death toll was got from the websites of the National Health Commission of the People’s Republic of China (http://www.nhc.gov.cn/). The daily average temperature and humidity data were obtained from Meteorological science data sharing service of China (http://data.cma.cn/site/index.html) and AQI data from air quality monitoring analysis online platform of China (https://www.aqistudy.cn/historydata/). The data of MSI and UTI were obtained from the Baidu map migration platform in the people’s Republic of China (https://qianxi.baidu.com/).

## Abbreviations

COVID-19: Coronavirus disease 2019
DLNM: Distributed lag non-linear model;
EHF: Ebola hemorrhagic fever
AIDS: Acquired Immune Deficiency Syndrome
AQI: Air quality index
SD: Standard deviation
Min: The minimum value
Max: The maximum value
P (25): Upper quartile
P (75): Lower quartile
RR: Relative risk
CI: Confidence interval
